# Clinical Impact of Breast Cancer Stem Cells in Metastatic Breast Cancer Patients

**DOI:** 10.1155/2020/2561726

**Published:** 2020-06-27

**Authors:** M. A. Elbaiomy, Tamer Akl, Nadia Atwan, Ahmed Ali Elsayed, Maha Elzaafarany, S. Shamaa

**Affiliations:** ^1^Medical Oncology Unit, Oncology Center, Mansoura Faculty of Medicine, Mansoura, Egypt; ^2^Pathology Department, Faculty of Medicine, Mansoura University, Mansoura, Egypt

## Abstract

**Background:**

Breast tumors are composed of phenotypically diverse groups of cells; however, it is unclear which of these cells contribute to tumor development. Breast cancer management usually targets proliferating cells, but as breast cancer stem cells are slowly cycling, they may escape these targets whenever they are not actively proliferating. This may explain the occurrence of recurrences and failure of the treatment.

**Aim:**

To assess the impact of the BCSC expression on progression-free survival (PFS), overall survival (OS), and tumor response in metastatic breast cancer patients and to correlate the BCSC expression with different clinicopathological parameters.

**Material:**

This prospective study enrolled 76 de novo metastatic breast cancer patients recruited from the Oncology Center, Mansoura University, Egypt, with a minimum age 31 years and a maximum of 70 years. Pretreatment BCSC markers (CD44 and CD24) were assessed by immunohistochemistry on formalin-fixed paraffin-embedded tumor tissues from a primary or metastatic site. Patients received different lines of treatment, hormonal or chemotherapy, according to their biological subtypes. Anti-Her2 was added for Her2-positive patients.

**Results:**

Thirty-three patients (43.4%) were premenopausal and 43 patients (56.6%) were postmenopausal. Bone-only metastasis was seen in 12 patients (15.7%), however, visceral ± bone metastasis was seen in 64 patients (84.3%). BCSC markers (CD44+ve and CD24−ve) were expressed in 32 patients (42.1%), while 44 patients (57.9%) were not expressing BCSC markers. Out of 32 patients expressing BCSC, 22 patients (68%) were premenopausal and 28 patients (87.5%) were with high-grade (GIII) disease. BCSC was significantly presented in triple negative subtype breast cancer as there were 32 patients with the BCSC expression, and out of them, 15 patients (46.9%) had triple negative disease, 10 patients (31.3%) had luminal subtype, and seven patients (21.9%) were Her2-amplified, while there were 44 patients without BCSC expression, and out of them, 30 patients (68.2%) were of the luminal subtype, no patient (20.5%) had triple negative disease, and five patients (11.4%) were Her2-amplified (P 0.006). Twenty-four patients (31.5%) presented with visceral crisis; out of them, 17 patients (70.1%) were expressing BCSC which also denoted more aggressive disease. Seventy-four patients were candidates for the response assessment. BCSC-expressing patients showed poor response compared to non-BCSC (16.1% responsive versus 51.2%, respectively), with a significance relation (*P* 0.003). The BCSC expression was associated with both significant short PFS (median, 18 months vs. 35 months; *P*=0.001) and short OS (median, 26 months vs. 43 months; *P*=0.003). In multivariate analysis; BCSC expression was an independent prognostic factor for poor OS (*P*=0.055) along with the molecular subtype (*P*=0.012), Her2 status (*P*=0.011), and histologic grade (*P*=0.037).

**Conclusion:**

This study further validates the BCSC expression as a poor prognostic biomarker correlated with poor response, short PFS and OS. So, it could be used as a marker for tailoring treatment with different lines of therapies in further studies. The BCSC expression was highly presented in the triple negative subtype which is an aggressive disease that lacks different targets. So, targeting BCSC may carry a hope in future for this group of patients.

## 1. Background

Cancer stem cells (CSCs) are responsible for cancer initiation, distant spread, and recurrence. It is known that tumors at different stages of the disease are compressed from heterogeneous cells [1] with different phenotypic patterns and variable proliferation capabilities. However, only the CSC population is in vitro and in vivo clonogenic and suggests the highest possible tumorigenic potential [2]. One of the main characteristics of the Cancer stem cells is their ability to control stemness pathways such as the Wnt/*β* catenin, Sonic Igel (Shh), and Transforming Growth Factor Beta (TGF-*β*) [3]. Both pathways are impaired in CSCs and have been used to enhance the effectiveness of treatment. The CSC model postulates a hierarchical system of solid tumors and leukemia, with CSCs at the top of this hierarchy, increasing tumor growth, relapse, and resistance to treatment [4].

Cell heterogeneity is responsible for differences in cell morphology, proliferative index, genetic changes, and treatment response [5]. For effective treatment, all CSCs should be explicitly removed to prevent the relapse of the tumor. Breast tumors are well known to be made up of phenotypically diverse groups of cells; however, it is not certain which of these types of cells contribute to tumor growth. In comparison to the theory that all cell populations have the potential to become tumorigenic by mutation accumulation, another hypothesis limits this propensity for a select community of cells that share the classic characteristics of stem cells, such as the ability to self-renew and differentiate [6].

New breast cancer therapy targets proliferating cells, but since the stem cells of breast cancer are circulating slowly, they will avoid targeted treatments because they do not deliberately proliferate. This may be one of the key factors behind breast cancer failures in management [2]. CD44 is a glycoprotein that has been involved in many cell functions such as cell adhesion, proliferation, signaling, migration, hematopoiesis, and activation of lymphocytes [7]. It functions as a receptor for many extracellular matrix components [8].

CD44 is widely used as a CSC marker, especially for epithelial tumors, and can be used either alone or in CD24 to identify breast CSCs [9]. In solid malignancy, CD44+ CD24−ve cells, in a variety of solid malignancies including breast cancer, have been classified as CSCs [10]. Aldehyde dehydrogenase (ALDH) is also a CSC marker and can be used for the identification of BCS cells in breast cancers [11].

During treatment, many cancers developed drug resistance which denotes that cancers have cells that are more resistant than the rest of the tumors. One of the most studied drug-resistance mechanisms in CSCs is their ability to actively expel therapeutic drugs through protein transport. These proteins are a family known as cassette carriers that bind ATP. These proteins act by ATP-dependent drug efflux pumps to extract the drug in the extracellular space and are found to be overexpressed in CSCs using side population assays [12].

## 2. Materials

**Figure 1:**
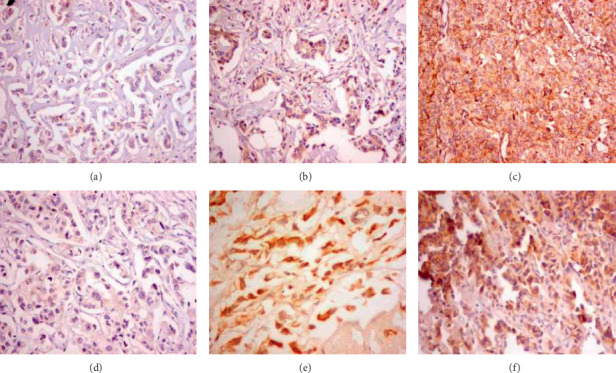
Different patterns of expressions (immunohistochemistry staining), CD44 and CD24, in the studied breast cancer tissues. (a) CD44−ve. (b) CD44+. (c) CD44++. (d) CD24−ve. (e) CD24+. (f) CD44++.

This prospective research enrolled 76 de novo metastatic breast cancer patients recruited from the Oncology Center, Mansoura University, Egypt, with a minimum age of 31 years and a maximum of 70 years. Pretreatment BCSC markers (CD44 & CD24) were evaluated by immunohistochemistry on formalin-fixed paraffin-embedded tumor tissues from primary or a metastatic site before treatment (sometimes tumor tissues not possible for biopsy or not representative after chemotherapy or radiotherapy). A distinct brown cytoplasmic immunostaining was scored positive. At least, 400 cells from 5 randomly selected fields (X400) were counted. Aberrant expression was defined as staining in excess of normal tissues. Three semiquantitative classes were used to describe the percentage of positively stained tumor cells; however, there was no common cutoff value on the patterns of staining. In this study, the grade was designated according to the following criteria: −ve negative, (+1) when the expression was in less than 50% of the tumor cells and (+2) when the expression was in more than 50% of cells. The expression was considered positive if (+) or (++) of the tumor cells were positively stained and negative otherwise, Figure 1.

Patients received different lines of treatment, hormonal or chemotherapy, according to their biological subtypes. For Her2-positive patients, anti-Her2 has been added. We aimed to evaluate the effect of the BCSC expression on PFS, OS, and tumor response in patients with metastatic breast cancer and compare the BCSC expression with various clinicopathological parameters.

The statistical analysis was carried out using software Excel 2007 and version 16 of SPSS (Statistical System for Social Science). Qualitative data were described in the form of numbers and percentages. Quantitative data were described in the form of mean (±) standard deviation (SD). Statistical analysis was performed by comparison between groups using the chi-squared test. The Kaplan–Meier product-limit estimator was used for calculating the survival measure. Comparison of the survival was performed by the log-rank test; continuous variables were dichotomized at the median cutoff. The probability of being by chance (*P* value) was calculated for all parameters (*P* is significant if <0.05 or =0.05 at a confidence interval of 95%).

## 3. Results

The study included 76 female patients with a minimum age of 31 years and a maximum of 70 years (median of 45.5 years). Thirty-three patients (43.4%) were premenopausal, and 43 patients (56.6%) were postmenopausal. Ten cases (13.2%) had bone-only metastasis, 19 cases (25%) had visceral-only metastasis, and 47 cases (61.8%) had both bone and visceral metastasis. BCSC markers (CD44+ve and CD24−ve) were expressed in 32 patients (42.1%), while 44 patients (57.9%) were not expressing BCSC markers, Table 1.

The age was significantly related to an increased BCSC expression as we found that patients with BCSC (CD44+ve and CD24−ve) were younger (mean ± SD; 43.6 ± 9.3) to the patients without the BCSC expression (mean ± SD; 49.9 ± 9.7) with *P* 0.006. Patients expressing BCSC (CD44+ve and CD24−ve) were more premenopausal (22 patients; 68.8%), while patients lacking the BSCS expression were more postmenopausal (23 patients; 52.3%) without a significant relation (*P* 0.1). The BSCS expression was significantly associated with high-grade tumor (28 patients grade III; 87.5% versus four patients grade II; 12.5%); however, patients not expressing BCSC had low-grade tumor (26 patients grade II; 59.1% versus 18 patients grade III; 40.9%) with P0.001. We did not found a relation between the BCS expression and the site of metastasis; however, there were 24 patients (31.5%) presenting with visceral crisis; out of them, 17 patients (70.1%) were expressing BCSC which also denoted more aggressive disease, see Table 2.

BCSC was significantly presented in triple negative subtype breast cancer as there were 32 patients with the BCSC expression, and out of them, 15 patients (46.9%) had triple negative disease, 10 patients (31.3%) had the luminal subtype, and seven patients (21.9%) were Her2-amplified, while 44 patients were without the BCSC expression, and out of them, 30 patients (68.2%) were of the luminal subtype, no patient (20.5%) had triple negative disease, and five patients (11.4%) were Her2-amplified with (*P* 0.006), see Table 2.

Seventy-four patients were candidates for the response assessment. BCSC-expressing patients showed a significant poor response to treatment; five patients (16.1%) were responders (either CR, PR or stable disease), and 26 patients (83.9%) were non-responders (progressed disease), while non-BCSC-expressing patients showed better response to treatment; 22 patients (51.2%) were responders (either CR, PR, or stable disease), and 21 patients (48.8%) were non-responders (progressed disease) with a significance relation (*P* 0.003), see Table 2.

**Figure 2:**
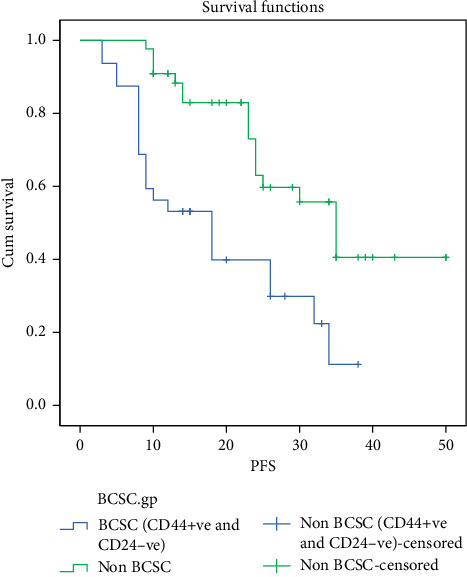
PFS of patients with the BCSC expression.

Patients with CD44+ve CD24−ve (BCSC) expression demonstrated an association with shorter PFS (median 18 months) in comparison with non-BCSC-expressing patients (median 35 months), and the difference was statistically significant (*P*=0.001), see Table 3 and Figure 2.

**Figure 3:**
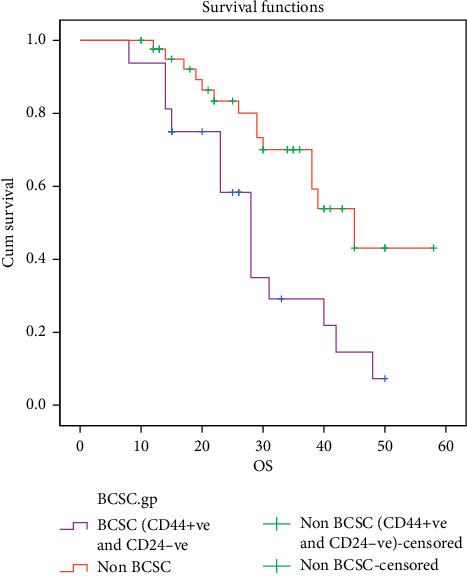
PFS of patients without the BCSC expression.

With a median follow-up duration of 25 months, patients with the BCSC expression (CD44+ve CD24−ve) demonstrated a significant poor OS (median 26 months) compared to patients without BCSC expression (median 43 months) with *P* 0.003, see Table 3 and Figure 3.

Multivariate analysis was performed by using the Cox proportional hazards model to determine whether BCSC has an independent prognostic value for OS or not. The BCSC expression was an independent prognostic factor for poor OS (*P*=0.055) along with the molecular subtype (*P*=0.012), Her2 status (*P*=0.011), and histologic grade (*P*=0.037), Table 4.

## 4. Discussion

BCSCs are characterized by self-renewal and the ability to differentiate, producing phenotypically diverse cells. There have been several pathways studied in BCSC self-renewal control including Notch, Hedgehog, and Wnt. In addition, many transcription factors control BCSCs such as NF-ÿB, c-Jun, Dach1 forkhead-like protein, and p21CIP1 for the CDK inhibitor [13]. BCSCs display cell motility and invasion and overexpress and promote metastasis genes [13].

CD44+ CD24−/low ESA+ (epithelial surface antigen, also known as EpCAM) cells were identified as CSCs in a number of solid malignancies such as breast cancer [10]. These cells are thought to share stem cell-like properties because they are capable of reconstituting the heterogeneity of the original primary tumors. CD44 is a useful marker for collecting CSCs not only in breast tumors but also in a variety of other tumor models [14]; CD44 may also be important in metastasis. Through the implantation of patient tumors or breast CSCs into mouse mammary fat pads and the use of noninvasive imaging strategies, it was demonstrated that CD44+ cells from both primary tumors and lung metastases showed high tumorigenicity [15]. Furthermore, the CD44+/CD24−/low cell population was able to reinitiate tumors in NOD/SCID mice and retained this ability after serial passages. Thus, these cells, which had the ability to self-renew and to differentiate and which displayed tumorigenic capacity, had CSC features [2].

BCSCs showed resistance to both chemotherapy and radiotherapy. Administering neoadjuvant chemotherapy to patients with breast cancer raises the proportion of CD44+/CD24−/low in tumor cells and improves mammary development in vitro [16]. Likewise, paclitaxel and epirubicin in breast tumors enriche ALDH + cells [17]. Radiotherapy also enhances the proportion of CD44+/CD24−/low in xenografts in mouse [18].

Treatment resistance in BCSCs is associated with self-renewal and cell-signaling changes, such as Notch, Wnt, Hedgehog, and HER-2. For example, Notch-1 overexpression showed chemotherapy resistance [19] and radioresistance [20] of BCSCs. Such findings may be due to Notch's ability to improve the survivin antiapoptotic gene or cycline D1 induction. Increased survivin levels can deregulate several mitotic checkpoints, leading to genetic instability and inhibiting apoptosis caused by radiation and drugs [19]. Since cycline D1 is also a downstream target for signaling Wnt, Stat3, *β*-catenin, and NF-ÿB, it may be a significant target for stem cell expansion given its tumorigenic properties and increased therapy resistance, and BCSCs have been involved in therapeutic relapse.

The BCSCs that survived the selective pressure exerted by therapy will transmit reduced sensitivity to their offspring, encourage the appearance of clinical resistance, and enable a more aggressive tumor to develop over time [21].

Two studies conducted by Abraham et al. [22] and Mylona et al. [23] using double-staining immunohistochemistry have examined the presence of CD44+/CD24− tumor cells in a breast cancer specimen and reported frequencies ranging between 0 and 80%, and these results were in agreement with our study that showed 42% prevalence of CD44+/CD24− tumor cells.

The current study noted that the patterns of expression of CD44 were mainly membranous, whereas CD24 was expressed predominantly in the cytoplasm which was in agreement with the findings of other reports [24, 25]. In another method of detection observed by Li et al., they used flow-cytometric analysis to quantify CD44+/CD24− cells, a method that may be influenced by the content of fibrous tissue when used with tumor samples that need to be disintegrated and that does not allow a distinction of labeled invasive tumor cells from noncancerous breast epithelia or residual in situ carcinoma cells [26].

We found that patients with the BCS expression significantly had high-grade tumor denoting that BCS cells could have an aggressive behavior. In contrast to our results, Kim et al. found that 44% of CD44+ CD24−tumors were grade 3 and 46% were grade 2. Also, Ahmed et al. [28] showed that 46.9% of CD44+/CD24−breast cancer cases were grade 2 and 42.9% were grade 3, while 10.2% were grade 1. These differences could be due to different patient demography and the study design as the last study was conducted on early breast cancer patients and not metastatic as our report.

This study demonstrated that the BCS cells expression was highly presented in triple negative disease, and this could explain the aggressiveness of this subtype and may open the way for targeting BCS cells to improve these patients' outcomes. Similarly, there was a significant difference in the frequency of the presence of BCSc, with 9 (60%) of 15 triple negative cases having the BCSc compared with 8 (26.7%) of 30 non-triple negative cases (*P*=0.0009) [25].

In a report produced by Lee and his colleges, a higher proportion of CD44+/CD24− tumour cells and ALDH1 positivity in prechemotherapy tissues of 92 breast cancer patients was correlated with higher histologic grade, oestrogen receptor (ER) negativity, high Ki-67 proliferation index, and the basal-like subtype of breast cancer [29].

Other reports also observed that the most common subtype associated with BCSCs is the basal subtype, contributing this to higher grade and aggressiveness of the tumors [30–32].

In contrast to our results, Ahmed et al. showed that the luminal subtype represents 83% of CD44+/CD24−cases, 6.4% was Her2-overexpressing, and 2.1% was triple negative were (*P* < 0.001). The difference can be attributed to inclusion criteria in that study as they included early breast cancer patients [28].

This study did not demonstrate the relation between the BCS expression and site of metastasis; however, Abraham et al. observed a high percentage of CD44+/CD24−/low tumor cells in primary tumors of patients with distant metastasis, particularly, osseous metastases [22]. This finding explained previously stresses the role of CD44 as a homing receptor for distant tissue compartments, a view that is in line with the CD44 expression being associated with cell motility through linking with putative actin-binding proteins [33], and the difference in our report could be explained by more visceral plus bone metastasis that could be attributed to the delay in seeking medical advice of patients in our region till they have more advanced disease with disseminated visceral metastasis.

Our study validates BCSC that could be a predictive marker for poor response to treatment in metastatic breast cancer patients. We reported that patients expressing BCS significantly were poor responders to chemotherapy (progressed disease); however, patients not expressing BCS showed a better response (CR, PR, or SD). Also, it is interesting to note that 14 patients (58.3%) out of 24 triple negative showed progressed disease, and the remaining 10 patients had a variable response rate either CR, PR, or SD, denoting that BCS cells could be used for tailoring treatment for this poor behavior subtype of breast cancer in further large studies.

Consistent with our observation, many other reports have shown that BCSCs are associated with tumor recurrence and radiation resistance [16, 34, 35].

In other reports, approximately 25% of patients with invasive breast carcinoma have Her2 amplification, and most of these patients eventually develop resistance to Herceptin. Several mechanisms, including the involvement of BCS, have been postulated to explain the resistance to Herceptin [36, 37] and this was in agreement with our results.

Many studies using neoadjuvant agents demonstrate an increase in the proportion of the BCS (CD44+/CD24−) in the residual cancer status after neoadjuvant treatment, suggesting that these cells may indeed be resistant to these therapies and escape current therapies to target the cancer stem cell component [16, 26].

Consistent with our study, another study that included fifty randomly selected cases of invasive breast carcinoma showed that tumors with CD44+/CD24−ve BCS cells had a higher rate of recurrence/metastasis (41.2% versus 28.6%, *P*=0.03) [25].

Phuc and colleagues performed a study in which the stem cells of breast cancer CD44+CD24− were isolated from breast tumors; the expression of CD44 was downregulated with siRNAs followed by treatment with specific antitumor concentrations. After treatment with the medication, the proliferation of downregulated CD44+CD24− breast cancer stem cells decreased. They noticed treated cells were more sensitive to doxorubicin, even at low doses, compared with the control groups [38].

In contrast, Alumann et al. found no association between the BCS cell expression and tumor response [39], and this contradictory result could be explained by the chemotherapy regimens and cycles that differed in the two studies, and there may be treatment-specific influences on outcomes.

The current study demonstrated that the BCS cells expression significantly had poor PFS and poor OS which could customize BCS cells as a prognostic marker for metastatic breast cancer.

Consistent with our results, Lee and colleges studied the impact of BCS cells on 92 breast cancer patients after chemotherapy and found that cases with increased BC cells or aldehyde dehydrogenase 1+ (ALDH1+) phenotypes (a reliable marker for breast cancer stem cells) had significantly shorter disease-free survival time, and they concluded that their study provided the clinical evidence that the BCS cells in breast cancer are chemoresistant and are associated with disease progression, emphasising the need for targeting BCS cells in breast cancer therapies [29].

Also, another study conducted in 203 primary breast cancer patients with ALDH1-positive tumors showed marginally significantly lower RFS (relapse-free survival) rates than those with ALDH1-negative tumors (*P*=0.056) [30], and this was in agreement with our report.

Expression of ALDH1 was determined in a retrospective series of 109 IBC (inflammatory breast cancer) patients, and the authors found the ALDH1 expression correlated with the development of distant metastasis and with decreased survival. With a median follow-up period of 67 months, the ALDH1 expression was significantly correlated with metastasis-free survival (MFS; *P*=0.01) as well as with tumor-specific survival (SS; *P*=0.03) [40].

Using a threshold of 25% CD44+/CD24–ve ovarian cancer cells found in ascites, patients with >25% CD44+/CD24−ve were significantly more likely to have recurrence (83 vs. 14%, *P*=0.003) and had shorter median progression-free survival (6 vs. 18 months, *P*=0.01) [41].

Mylona and colleagues [23] reported that the prevalence of CD44+/CD24−ve phenotype had no prognostic value. In contrast, our study showed increased recurrence and poor survival in patients with the CD44+/CD24−ve phenotype. These contradictory observations in these 2 studies may be related to the cutoff used in defining CD44+/CD24−ve in the 2 studies and the study design. Of note, Mylona and colleagues did not use any percentage or intensity cutoff in their studies, stating that “CD44 was identified as black and CD24 as red membrane staining. Cells with black color staining without much interference from the red color were identified as CD44+/CD24−ve tumor cells.”

In the study conducted by Alumann and his colleges, no association was found with progression-free or overall survival for the tumors containing CD44+/CD24− cells [39]. This result was in contrast to our results that showed significantly shorter PFS and OS associated with CD44+/CD24−cells, and this can be explained by alternative differences in immunostaining methods and scoring protocols.

The study conducted by Abraham et al. using the immunohistochemistry double immune staining technique on parrafin-embedded tissues of 136 patients showed no association of the percentages of CD44+/CD24−/low cells in tumors with response, and moreover, they had no effect on event-free or overall survival [22]. Although Abraham and colleagues described the percentages of CD44+/CD24−/low, it is unclear what percentage they used as the cutoff and whether the intensity of the staining was considered.

## 5. Conclusions

This study further validates the BCSC expression as a clinically useful marker for the identification of biologically aggressive breast cancers. We have shown that the expression of BCS cells in tumor tissues is a poor prognostic biomarker associated with poor response, limited PFS, and OS. So, it could be used as a marker in further studies to customize care with different therapy lines. The expression of BCSC was strongly presented in a triple negative subtype that is an aggressive disease lacking specific targets. So, targeting BCS cells may carry a hope in the future for this group of patients.

## Figures and Tables

**Table 1:** Demographic data of the studied cases.

Age (years)	Median	45.5
	Range	31─70
	Median	50

Family history	Positive family history	3 (4%)
	No family history	73 (96%)

Menopausal status	Premenopausal	33 (43.4%)
	Postmenopausal	43 (56.6%)
Metastatic site	Bone only	10 (13.2%)
	Visceral only	19 (25%)
	Bone and visceral	47 (61.8%)

BCSC expression	BCSC (CD44+ve and CD24−e) 32 patients (42.1%)	Non-BCSC 44 patients (57.9%)

**Table 2:** Patients' characteristics with the BCSC expression.

	BCSC (CD44+ve and CD24−ve) 32 patients	Non-BCSC 44 patients	*P*
Premenopausal	22 (68.8%)	21 (47.7%)	0.1
Postmenopausal	10 (31.2%)	23 (52.3%)	

Grade II	4 (12.5%)	26 (59.1%)	0.001
Grade III	28 (87.5%)	18 (40.9%)	

Luminal A and B	10 (31.2%)	30 (68.2%)	0.006
Her2-amplified	7 (21.9%)	5 (11.4%)	
Triple negative	15 (46.9%)	9 (20.4%)	

CR, PR, and SD	5 (16.1%)	22 (51.2%)	0.003
Progressed disease	26 (83.9%)	21 (48.8%)	

**Table 3:** PFS and OS of patients in relation to the BCSC expression.

	BCSC (CD44+ve and CD24−ve) 32 patients	Non-BCSC 44 patients	*P*
*PFS*			0.001
Median	18	35	
95% confidence interval (CI)	9–26.9	28.6–41.3	

*OS*			0.003
Median	26	43	
95% confidence interval (CI)	23.5–32.4	34.2–55.7	

**Table 4:** Multivariate analysis of OS.

Prognostic factors	Hazard ratio	95% confidence interval (CI)	*P* value
BCSCs			0.055
BCSCs	0.34	0.1─1.1	
Non-BCSCs	1		

Molecular subtype			0.012
Luminal A and B	1	1.2─6.9	
Her2 and basal	2.96		

Her2 disease		1.3─12.3	0.011
Negative disease	1		
Positive disease	4.13		

Grade			0.037
Low (G II)	1	1.05─6.09	
High (G III)	2.53		

## Data Availability

All data are available upon request to the corresponding author.

## References

[1] Dalerba P, Cho R. W., Clarke M. F. (2007). Cancer stem cells: models and concepts. *Annual Review of Medicine*.

[2] Al-Hajj M., Wicha M. S., Benito-Hernandez A., Morrison S. J., Clarke M. F. (2003). Prospective identification of tumorigenic breast cancer cells. *Proceedings of the National Academy of Sciences*.

[3] Ajani J. A. (2015). Cancer stem cells: the promise and the potential. *Seminars in Oncology*.

[4] Vlashi E., Pajonk F. (2015). Cancer stem cells, cancer cell plasticity and radiation therapy. *Seminars in Cancer Biology*.

[5] Visvader J. E. (2011). Cells of origin in cancer. *Nature*.

[6] Reya T., Morrison S. J., Clarke M. F., Weissman I. L. (2001). Stem cells, cancer, and cancer stem cells. *Nature*.

[7] Nosrati A., Naghshvar F., Khanari S. (2014). Cancer stem cell markers CD44, CD133 in primary gastric adenocarcinoma. *International Journal of Molecular and Cellular Medicine*.

[8] Yan Y., Zuo X., Wei D. (2015). Concise review: emerging role of CD44 in cancer stem cells: a promising biomarker and therapeutic target. *Stem Cells Translational Medicine*.

[9] Park C., Bergsagel D., McCulloch E. (1971). Mouse myeloma tumor stem cells: a primary cell culture assay2. *Journal of the National Cancer Institute*.

[10] Fillmore C. M., Kuperwasser C. (2008). Human breast cancer cell lines contain stem-like cells that self-renew, give rise to phenotypically diverse progeny and survive chemotherapy. *Breast Cancer Research*.

[11] Ginestier C., Hur M. H., Charafe-Jauffret E. (2007). ALDH1 is a marker of normal and malignant human mammary stem cells and a predictor of poor clinical outcome. *Cell Stem Cell*.

[12] Eyre R., Harvey I., Stemke-Hale K., Lennard T. W. J., Tyson-Capper A., Meeson A. P. (2014). Reversing paclitaxel resistance in ovarian cancer cells via inhibition of the ABCB1 expressing side population. *Tumor Biology*.

[13] Liu H. (2010). Cancer stem cells from human breast tumors are involved in spontaneous metastases in orthotopic mouse models. *Proceedings of the National Academy of Sciences*.

[14] Wei X., Dombkowski D., Meirelles K. (2010). Mullerian inhibiting substance preferentially inhibits stem/progenitors in human ovarian cancer cell lines compared with chemotherapeutics. *Proceedings of the National Academy of Sciences*.

[15] Liu H., Patel M. R., Prescher J. A. (2010). Cancer stem cells from human breast tumors are involved in spontaneous metastases in orthotopic mouse models. *Proceedings of the National Academy of Sciences*.

[16] Yu F., Yao H., Zhu P. (2007). let-7 regulates self renewal and tumorigenicity of breast cancer cells. *Cell*.

[17] Tanei T., Morimoto K., Shimazu K. (2009). Association of breast cancer stem cells identified by aldehyde dehydrogenase 1 expression with resistance to sequential Paclitaxel and epirubicin-based chemotherapy for breast cancers. *Clinical Cancer Research*.

[18] Velasco-Velázquez M. A., Homsi N., De La Fuente M., Pestell R. G. (2012). Breast cancer stem cells. *The International Journal of Biochemistry & Cell Biology*.

[19] Sajithlal G. B., Rothermund K., Zhang F. (2010). Permanently blocked stem cells derived from breast cancer cell lines. *Stem Cells*.

[20] Phillips T. M., McBride W. H., Pajonk F. (2006). The response of CD24−/low/CD44+ breast cancer-initiating cells to radiation. *JNCI: Journal of the National Cancer Institute*.

[21] Velasco-Velázquez M. A., Li Z., Casimiro M., Loro E., Homsi N., Pestell R. G. (2011). Examining the role of cyclin D1 in breast cancer. *Future Oncology*.

[22] Abraham B. K. (2005). Prevalence of CD44+/CD24−/low cells in breast cancer may not be associated with clinical outcome but may favor distant metastasis. *Clinical Cancer Research*.

[23] Mylona E., Giannopoulou I., Fasomytakis E. (2008). The clinicopathologic and prognostic significance of CD44+/CD24−/low and CD44−/CD24+ tumor cells in invasive breast carcinomas. *Human Pathology*.

[24] Bernardi M., Logullo A. F, Pasini F. S (2012). Prognostic significance of CD24 and claudin-7 immunoexpression in ductal invasive breast cancer. *Oncology Reports*.

[25] Idowu M. O., Kmieciak M., Dumur C. (2012). CD44+/CD24−/low cancer stem/progenitor cells are more abundant in triple-negative invasive breast carcinoma phenotype and are associated with poor outcome. *Human Pathology*.

[26] Li X., Lewis M. T., Huang J. (2008). Intrinsic resistance of tumorigenic breast cancer cells to chemotherapy. *JNCI Journal of the National Cancer Institute*.

[27] Kim H. J., Kim M.-J., Ahn S. H. (2011). Different prognostic significance of CD24 and CD44 expression in breast cancer according to hormone receptor status. *The Breast*.

[28] Ahmed M. A. H., Aleskandarany M. A., Rakha E. A. (2012). A CD44−/CD24+ phenotype is a poor prognostic marker in early invasive breast cancer. *Breast Cancer Research and Treatment*.

[29] Lee H. E., Kim J. H., Kim Y. J. (2011). An increase in cancer stem cell population after primary systemic therapy is a poor prognostic factor in breast cancer. *British Journal of Cancer*.

[30] Morimoto K., Kim S. J., Tanei T. (2009). Stem cell marker aldehyde dehydrogenase 1-positive breast cancers are characterized by negative estrogen receptor, positive human epidermal growth factor receptor type 2, and high Ki67 expression. *Cancer Science*.

[31] Nalwoga H., Arnes J. B., Wabinga H., Akslen L. A. (2010). Expression of aldehyde dehydrogenase 1 (ALDH1) is associated with basal-like markers and features of aggressive tumours in African breast cancer. *British Journal of Cancer*.

[32] Park S. Y., Lee H. E., Li H., Shipitsin M., Gelman R., Polyak K. (2010). Heterogeneity for stem cell–related markers according to tumor subtype and histologic stage in breast cancer. *Clinical Cancer Research*.

[33] Zohar R., Suzuki N., Suzuki K. (2000). Intracellular osteopontin is an integral component of the CD44-ERM complex involved in cell migration. *Journal of Cellular Physiology*.

[34] Horwitz K. B. (2008). Commentary: the year in basic science: update of estrogen plus progestin therapy for menopausal hormone replacement implicating stem cells in the increased breast cancer risk. *Molecular Endocrinology*.

[35] Kakarala M., Wicha M. S. (2008). Implications of the cancer stem-cell hypothesis for breast cancer prevention and therapy. *Journal of Clinical Oncology*.

[36] Bedard P., de Azambuja E., Cardoso F. (2009). Beyond trastuzumab: overcoming resistance to targeted HER-2 therapy in breast cancer. *Current Cancer Drug Targets*.

[37] Reim F., Dombrowski Y., Ritter C. (2009). Immunoselection of breast and ovarian cancer cells with Trastuzumab and natural killer cells: selective escape of CD44high/CD24low/HER2low breast cancer stem cells. *Cancer research*.

[38] Phuc P. V., Chinh Nhan P. L., Nhung T. H. (2011). Downregulation of CD44 reduces doxorubicin resistance of CD44+CD24− breast cancer cells. *OncoTargets and Therapy*.

[39] Aulmann S., Waldburger N., Penzel R., Andrulis M., Schirmacher P., Sinn H. P. (2010). Reduction of CD44+/CD24− breast cancer cells by conventional cytotoxic chemotherapy. *Human Pathology*.

[40] Charafe-Jauffret E., Ginestier C., Iovino F. (2010). Aldehyde dehydrogenase 1-positive cancer stem cells mediate metastasis and poor clinical outcome in inflammatory breast cancer. *Clinical Cancer Research*.

[41] Meng E., Long B., Sullivan P. (2012). CD44+/CD24− ovarian cancer cells demonstrate cancer stem cell properties and correlate to survival. *Clinical & Experimental Metastasis*.

